# Multiscale Quality Deterioration of Giant Freshwater Prawn (*Macrobrachium rosenbergii*) During Frozen Storage

**DOI:** 10.3390/foods15142436

**Published:** 2026-07-09

**Authors:** Hao Lu, Si Xu, Siyi Zhu, Runyang Lv, Xingxing Deng, Songyi Lin, Zhiqiang Lu

**Affiliations:** 1 College of Food Science and Pharmaceutics, Xinjiang Agricultural University, Urumqi 830002, China; 13315270015@163.com (H.L.); 17822741766@163.com (S.X.); 15071213881@163.com (S.Z.); 13332113626@163.com (R.L.); m13029617289@163.com (X.D.); 2 Xinjiang Tacheng Food and Drug Inspection Institute, Tacheng 834700, China; 3 National Engineering Research Center of Seafood, School of Food Science and Technology, Dalian Polytechnic University, Dalian 116034, China; linsongyi730@163.com

**Keywords:** giant freshwater prawn, frozen storage, water-holding capacity, water redistribution, protein oxidation, myofibrillar structure

## Abstract

Frozen storage is widely used to preserve shrimp products, but the multiscale mechanisms underlying quality deterioration in giant freshwater prawn remain insufficiently understood. This study investigated changes in water retention, oxidative stability, muscle structure, and protein conformation in *Macrobrachium rosenbergii* stored at −20 ± 2 °C for 0, 1, 3, and 5 months. Thawing and cooking losses, water-holding capacity, freshness and oxidation indices, texture, histology, low-field nuclear magnetic resonance, Fourier transform infrared spectroscopy, and intrinsic fluorescence were comprehensively evaluated. After 5 months, thawing loss increased from 8.21% to 12.51%, cooking loss increased by 55.0%, and water-holding capacity decreased from 85.45% to 68.46%. The total volatile basic nitrogen, protein carbonyl content, thiobarbituric acid reactive substances (TBARS; 0.186 to 0.528 mg MDA/kg), and myofibril fragmentation index increased progressively, while free sulfhydryl and salt-soluble protein contents declined. Shear force, hardness, cohesiveness, gumminess, and chewiness also decreased, accompanied by muscle fiber separation and an increase in white void area from 0.04% to 3.46%. Low-field nuclear magnetic resonance revealed decreases in the short-relaxation P_2_b and P_2_1 populations and an increase in the dominant P_2_2 population, indicating relative water-population redistribution. Meanwhile, α-helix content decreased from 18.83% to 16.28%, β-sheet content increased from 25.05% to 28.22%, and maximum fluorescence intensity decreased by 31.9%. These coordinated changes suggest that prolonged frozen storage weakened the myofibrillar network through water redistribution, lipid and protein oxidation, protein fragmentation, conformational rearrangements, and tissue disruption. The findings provide a multiscale basis for developing water-retention and quality-control strategies for frozen giant freshwater prawn.

## 1. Introduction

As an important freshwater aquaculture species, the giant freshwater prawn (*Macrobrachium rosenbergii*) is highly appreciated for its sensory quality and nutritional value. However, prawn muscle is highly susceptible to postmortem quality deterioration because of its high moisture content, delicate tissue structure, and active endogenous enzymes. Although frozen preservation helps maintain the supply and extend the usable life of shrimp and prawn products, it cannot fully arrest the physicochemical and structural changes responsible for quality deterioration. During prolonged frozen storage, ice-crystal formation and recrystallization can damage cell membranes and muscle fibers, enlarge inter-fiber spaces, and disrupt the myofibrillar network, thereby increasing thawing loss, cooking loss, and texture deterioration [1,2,3]. In parallel, oxidation and denaturation of myofibrillar proteins, together with possible conformational rearrangements, can weaken protein-water interactions and reduce the ability of the muscle network to immobilize water [4,5]. Because water-holding capacity directly affects processing yield, eating quality, and consumer acceptance, characterizing the concurrent changes in water-population distribution, protein deterioration, and microstructural damage is essential for understanding quality loss in frozen prawn muscle.

A range of physicochemical indicators has been used to evaluate the deterioration of frozen aquatic products. TVB-N is generally used to indicate the accumulation of volatile nitrogenous degradation products, while TBARS serves as an index of secondary products generated during lipid oxidation. Protein carbonyl and free sulfhydryl contents provide information on protein oxidation, while salt-soluble protein content and the myofibril fragmentation index reflect protein extractability and myofibrillar integrity [2,4]. However, these conventional indicators alone provide limited information on the structural basis of water loss. Low-field nuclear magnetic resonance (LF-NMR) can characterize the relative distribution of water populations, while magnetic resonance imaging provides spatial visualization of water-related signals. Fourier transform infrared spectroscopy (FTIR) and intrinsic fluorescence spectroscopy can further reveal changes in protein secondary and tertiary structures, respectively. In addition, histological staining enables direct observation and quantitative analysis of muscle-fiber arrangement, connective tissue, and inter-fiber voids. Integrating these complementary approaches may therefore provide a more comprehensive understanding of frozen-storage-induced deterioration across physicochemical, molecular, and tissue levels [6,7].

Previous studies have examined the effects of freezing methods, frozen storage, and repeated freeze–thaw cycles on the quality of shrimp and other aquatic products. Boonsumrej et al. [1] reported that freezing and thawing increased drip loss and damaged the muscle structure of tiger shrimp. Sriket et al. [2] further showed that repeated freeze–thaw cycles induced protein denaturation, cellular disruption, and microstructural deterioration in black tiger shrimp and Pacific white shrimp. For giant freshwater prawn, Yu et al. [3] compared different freezing methods during frozen storage and reported changes in freshness, thawing loss, texture, and muscle microstructure. These studies provide important evidence that freezing conditions strongly influence the quality and stability of prawn and shrimp muscle. However, previous studies on giant freshwater prawn have mainly focused on comparisons of freezing methods or conventional quality evaluation, while coordinated changes in water retention, lipid and protein oxidation, myofibrillar integrity, tissue-level structural disruption, water-population redistribution, and protein secondary and tertiary conformations during prolonged frozen storage remain insufficiently characterized. In particular, the integration of LF-NMR, quantitative histological analysis, FTIR, and intrinsic fluorescence spectroscopy with conventional freshness, oxidation, and texture indicators has rarely been applied to the giant freshwater prawn. Consequently, the structural and molecular basis underlying the loss of water-holding capacity and texture in frozen giant freshwater prawn remains insufficiently characterized.

Accordingly, this study aimed to characterize the deterioration of giant freshwater prawn muscle during 5 months of frozen storage at −20 °C and to provide an integrated evaluation of coordinated changes in water-population distribution, lipid and protein oxidation, protein conformation, and muscle-tissue structure. Thawing behavior, thawing and cooking losses, water-holding capacity, freshness-related indices, lipid and protein oxidation, salt-soluble protein content, myofibril fragmentation index, and textural properties were determined. Masson’s trichrome staining, LF-NMR and magnetic resonance imaging, FTIR, and intrinsic fluorescence spectroscopy were further employed to characterize changes in muscle microstructure, water distribution, and protein secondary and tertiary conformations. The findings provide a multiscale understanding of frozen-storage-induced quality deterioration and may support the development of quality-control and water-retention strategies for frozen prawn products.

## 2. Materials and Methods

### 2.1. Materials

Fresh giant freshwater prawns (*Macrobrachium rosenbergii*) of similar size and without visible mechanical damage were purchased from Beiyuanchun Market in Urumqi, Xinjiang, China, between September and October. The giant freshwater prawns were purchased as freshly deceased food products intended for human consumption, and all experiments were conducted exclusively on postmortem muscle tissues. All prawns were artificially cultured for approximately 5–6 months and were obtained from a single commercial batch. The average body weight of the prawns was 38 ± 3 g, and the body length ranged from 11 to 12 cm. The prawns were transported to the laboratory in insulated boxes with crushed ice and processed immediately upon arrival. After removing the head, shell, and intestinal tract, the abdominal muscle was collected as peeled prawn samples, rinsed with chilled distilled water, drained, and randomly divided into four groups. The peeled prawns were packed in polyethylene pouches, with three individual peeled prawns per pouch, and arranged as a single layer. The pouches were sealed without vacuum treatment and stored in a conventional laboratory freezer at −20 ± 2 °C. The freezing rate and the time required for the sample core temperature to reach −20 °C were not directly measured. The samples were analyzed after 0, 1, 3, and 5 months. For each storage period, three individual prawns were randomly selected for each measurement, and each individual prawn was treated as one independent biological replicate (*n* = 3). No individual prawn was shared among different storage groups. The 0-month samples were analyzed after the initial freezing–thawing procedure without further frozen storage. Thus, the 0-month group represented an initial frozen–thawed control rather than fresh, unfrozen prawn muscle. Before analysis, frozen samples were thawed naturally at room temperature until the core temperature of 4 °C was reached.

### 2.2. Determination of Thawing Time

Peeled prawn samples were divided into portions of 100 ± 10 g, placed individually in polyethylene bags, and stored at −20 ± 2 °C for the designated storage periods. At the specified storage intervals, the packaged samples were transferred from the freezer and thawed under ambient laboratory conditions. During thawing, the ambient temperature was maintained at 20–25 °C, and the relative humidity was approximately 45–50%. Thawing was performed on a laboratory bench under still-air conditions, and no forced-air circulation was applied. The temperature probe of a temperature and humidity data logger (Elitech GSP-6Pro, Xuzhou, China) was inserted into the geometric center of each packaged sample. The core temperature was recorded at 10 min intervals throughout the thawing process. Thawing time was defined as the elapsed time from removal of the sample until its core temperature reached 4 °C.

### 2.3. Determination of Thawing Loss

Thawing loss was determined using a modified procedure based on Kong et al. [8]. The initial mass of each peeled prawn sample was recorded before frozen storage. At each designated storage interval, the samples were thawed at ambient laboratory temperature until the core temperature reached 4 °C. The exudate was removed, and surface moisture was gently blotted with absorbent paper before the samples were reweighed. Thawing loss was expressed as the percentage reduction in sample mass after thawing relative to the initial mass before frozen storage.

### 2.4. Determination of Cooking Loss

Cooking loss was determined according to Kong et al. [8], with minor modifications. After thawing to a core temperature of 4 °C, the peeled prawn samples were gently blotted with absorbent paper to remove surface moisture and weighed before cooking. Each sample was transferred to a heat-resistant pouch, sealed, and cooked in a water bath maintained at 80 °C for 30 min. After cooking, the samples were removed from the bags, cooled to room temperature, and gently blotted to remove surface moisture before being reweighed. Cooking loss was expressed as the percentage reduction in sample mass after cooking relative to the mass before cooking.

### 2.5. Determination of Water-Holding Capacity

Water-holding capacity was evaluated using a previously reported method Guan et al. [9], with minor modifications. After thawing to a core temperature of 4 °C, the surface moisture of the peeled prawn samples was gently removed using absorbent paper. Samples of uniform size were cut from the abdominal region and weighed before centrifugation. Each sample was wrapped in filter paper, placed in a centrifuge tube, and centrifuged at 5030× *g* for 10 min at 4 °C. After centrifugation, the samples were removed from the filter paper and reweighed. Water-holding capacity was expressed as the percentage of the sample mass retained after centrifugation relative to its mass before centrifugation.

### 2.6. Determination of Total Volatile Basic Nitrogen

A modified version of the method described by Zhu et al. [10] was used to quantify the total volatile basic nitrogen (TVB-N). A 5.0 g portion of minced, peeled prawn muscle was combined with 50 mL of distilled water in a conical flask and agitated for 30 min to obtain the extract. The mixture was then filtered, and 5 mL of the filtrate was mixed with a magnesium oxide suspension (10 g/L). Steam distillation was performed for 5 min using an automatic Kjeldahl nitrogen analyzer (ATN-300, Hongji Instrument Ltd., Shanghai, China). The volatile basic compounds were collected in a boric acid solution and titrated to the endpoint with 0.01 mol/L standardized hydrochloric acid solution. The TVB-N content was expressed as mg N/100 g of sample.

### 2.7. Determination of Protein Carbonyl and Free Sulfhydryl Contents

Protein carbonyl and free sulfhydryl contents were determined using commercial assay kits strictly according to the manufacturer’s instructions. Briefly, minced peeled prawn muscle was used as the starting material and processed with the reagent/extraction solution supplied with the corresponding assay kit under chilled conditions. The assay solutions were prepared from prawn muscle homogenates according to the kit protocols, rather than from independently extracted or purified protein fractions. No additional protein extraction or purification procedure was performed before the measurements. Protein concentration, blank controls, calibration standards, incubation conditions, and absorbance measurements were all conducted according to the corresponding kit protocols. Protein carbonyl content was measured using a Protein Carbonyl Content Assay Kit (BC1270, Solarbio Science & Technology Co., Ltd., Beijing, China). The absorbance of the 2,4-dinitrophenylhydrazine-derived reaction product was measured at 370 nm using a UV-visible spectrophotometer, and the results were expressed as nmol/mg protein. Free sulfhydryl content was determined using a Protein Free Thiol Content Assay Kit (BC5895, Solarbio Science & Technology Co., Ltd., Beijing, China). The reaction between protein sulfhydryl groups and 5,5′-dithiobis-(2-nitrobenzoic acid) was measured at 412 nm using a microplate reader.

### 2.8. Texture Profile Analysis and Shear Force Measurement

Texture properties were determined according to Huo et al. [11], with minor modifications. After thawing to a core temperature of 4 °C, the surface moisture of the peeled prawns was removed using absorbent paper. Texture measurements were performed on raw, thawed, peeled prawn muscle rather than cooked muscle. The whole peeled abdominal muscle was used as the test sample without being cut into fixed-size blocks, and each sample was placed horizontally on the testing platform. Measurements were conducted at room temperature (20–25 °C). For each biological replicate, measurements were performed at three different positions of the abdominal muscle, and the average value was used for statistical analysis. Texture profile analysis was performed using a TA.XTplus texture analyzer (Stable Micro Systems Ltd., Godalming, UK), equipped with a cylindrical probe (P/36R, 36 mm diameter). Each sample was placed horizontally on the testing platform and compressed twice to 50% of its original height. The trigger force was 5 g, and the pre-test, test, and post-test speeds were set at 2, 1, and 1 mm/s, respectively. Hardness, springiness, cohesiveness, gumminess, and chewiness were recorded. For shear force measurement, the sample was cut perpendicular to the longitudinal direction of the muscle fibers using a blade-type probe. The instrument was operated at pre-test, test, and post-test speeds of 2, 1, and 2 mm/s, respectively, and the maximum force during cutting was recorded as the shear force.

### 2.9. Determination of Myofibril Fragmentation Index

The myofibril fragmentation index (MFI) was determined according to Huo et al. [11], using a slightly modified version. Minced prawn muscle (2.00 g) was homogenized for 1 min with 20 volumes of precooled MFI buffer containing 100 mmol/L KCl, 11.2 mmol/L K_2_HPO_4_, 8.8 mmol/L KH_2_PO_4_, 1 mmol/L EGTA, and 1 mmol/L MgCl_2_. The resulting homogenate was passed through three layers of gauze, followed by centrifugation at 11,000× *g* for 10 min at 4 °C. The pellet was washed once under the same conditions and resuspended in MFI buffer. After adjusting the protein concentration to 0.50 ± 0.05 mg/mL using the biuret method, the absorbance was measured at 540 nm. MFI was calculated as A_540_ × 200.

### 2.10. Determination of Salt-Soluble Protein Content

Salt-soluble protein content was determined according to Zhu et al. [10], with minor modifications. Minced peeled prawn samples (2.00 g) were homogenized with 20 mL of precooled 0.6 mol/L KCl solution (pH 7.0). The homogenate was extracted at 4 °C for 1 h and then centrifuged at 8000× *g* for 10 min. The protein concentration in the supernatant was determined using the biuret method. Salt-soluble protein content was calculated from the protein concentration and total extract volume relative to the sample mass and expressed as mg/g sample.

### 2.11. Measurement of Thiobarbituric Acid Reactive Substances

Thiobarbituric acid-reactive substances (TBARSs) were determined according to Zhu et al. [10], with minor modifications. Minced peeled prawn samples (5.0 g) were homogenized with 20 mL of trichloroacetic acid extraction solution containing 37.5 g of trichloroacetic acid and 0.5 g of disodium ethylenediaminetetraacetate per 500 mL of distilled water. The homogenate was centrifuged at 8000× *g* for 10 min at 4 °C. Subsequently, 5 mL of the supernatant was mixed with 5 mL of 0.02 mol/L thiobarbituric acid solution and incubated in a water bath at 90 °C for 30 min. The reaction mixture was cooled to room temperature under running water, and the absorbance was measured at 532 nm using an xMark microplate absorbance spectrophotometer (Bio-Rad Laboratories, Inc., Hercules, CA, USA). The extraction solution was used as the blank, and 1,1,3,3-tetraethoxypropane was used to construct the standard curve. The TBARS value was expressed as mg malondialdehyde/kg of sample.

### 2.12. Masson’s Trichrome Staining and Histological Analysis

Masson’s trichrome staining was performed according to Huo et al. [11], with minor modifications. Muscle blocks were collected from the same abdominal region of three independent prawns in each storage group and processed for paraffin sectioning and Masson’s trichrome staining. For each biological replicate, three non-overlapping fields were randomly selected from one representative section and analyzed using ImageJ software (version 1.54g, National Institutes of Health, Bethesda, MD, USA). The relative area fractions of red-stained muscle fibers, blue-stained connective tissue, and white voids were quantified using color threshold segmentation. The same threshold settings were applied to all images to select the corresponding stained regions. The blue-stained connective tissue area ratio was calculated as the blue-stained area divided by the total tissue area and expressed as a percentage. The values obtained from the three fields were averaged to generate one value for each independent sample. Thus, three biological replicate values were used for subsequent statistical analysis (*n* = 3).

### 2.13. Low-Field Nuclear Magnetic Resonance Measurements

Water distribution and mobility in peeled prawns were analyzed using a low-field nuclear magnetic resonance analyzer (NMI20-030H-1, Niumag Analytical Instrument Co., Ltd., Suzhou, China), according to Liang et al. [12] with modifications. For each storage period, three independent thawed peeled prawns were analyzed as biological replicates (*n* = 3), and each replicate represented a separate individual prawn. Each biological replicate was measured once under the same LF-NMR parameters, and the eight repeated scans were used for signal accumulation rather than treated as independent replicates. The Carr–Purcell–Meiboom–Gill pulse sequence was used. The durations of the 90° and 180° pulses were set to 23 and 42 μs, respectively. A total of 1500 echoes were collected with eight repeated scans, and the repetition time was 3000 ms. The transverse relaxation data were analyzed using MultiExp Inv Analysis software (v8.3.2, Suzhou Niumag Analytical Instrument Co., Ltd., Suzhou, China). Water components within 0.1–1, 1–10, and 10–100 ms were assigned as T_2_b, T_2_1, and T_2_2, respectively. The relative peak-area proportions corresponding to T_2_b, T_2_1, and T_2_2 were denoted as P_2_b, P_2_1, and P_2_2, respectively.

### 2.14. Fourier Transform Infrared Spectroscopy

Protein secondary structure was analyzed using Fourier transform infrared spectroscopy according to Huo et al. [11], with minor modifications. Salt-soluble muscle protein extracts were prepared using the same extraction procedure as described for intrinsic fluorescence spectroscopy. Briefly, thawed peeled prawn muscle samples were minced, homogenized with precooled 0.6 mol/L KCl solution (pH 7.0), and extracted at 4 °C for 1 h. The homogenate was centrifuged at 8000× *g* for 10 min at 4 °C, and the supernatant was collected as the salt-soluble muscle protein extract. The obtained protein extract was freeze-dried before FTIR analysis. FTIR spectra were collected using the KBr pellet method with an FTIR spectrometer (Nicolet iS10, Thermo Fisher Scientific Inc., Waltham, MA, USA). Briefly, the freeze-dried protein sample was mixed with dry spectroscopic-grade KBr, ground thoroughly, and pressed into a transparent pellet for spectral acquisition. Spectra were collected over the range of 4000–400 cm^−1^ at a resolution of 4 cm^−1^ with 32 accumulated scans. A background spectrum was recorded before each measurement. The amide I region (1600–1700 cm^−1^) was subjected to baseline correction and multi-peak fitting using OriginPro 2024 software. The relative contents of α-helix, β-sheet, β-turn, and random-coil structures were calculated from the area of each fitted peak as a percentage of the total amide I peak area.

### 2.15. Intrinsic Fluorescence Spectroscopy

Intrinsic fluorescence spectra were measured according to Huo et al. [11], using a slightly modified version. Minced peeled prawn samples were homogenized with precooled 0.6 mol/L KCl solution (pH 7.0) and extracted at 4 °C for 1 h. The homogenate was centrifuged at 8000× *g* for 10 min at 4 °C, and the supernatant was collected as the salt-soluble muscle protein extract. The protein concentration of the supernatant was determined using the biuret method and adjusted to 1.0 mg/mL with the same extraction buffer. The extracted prawn muscle protein solution was analyzed using an FLS1000 fluorescence spectrometer (Edinburgh Instruments Ltd., Livingston, UK). Fluorescence emission was scanned over 300–450 nm using an excitation wavelength of 280 nm, with both monochromator slit widths fixed at 1 nm. The maximum emission wavelength (λmax), maximum fluorescence intensity (Imax), and integrated peak area were determined using OriginPro 2021 software.

### 2.16. Statistical Analysis

All experiments were performed using three independent biological replicates for each storage period and each measurement. Each biological replicate represented one individual prawn, and no pooled samples were used. When repeated readings or multiple technical measurements were performed within the same biological replicate, the average value was first calculated and then used as the value for that biological replicate. Technical replicates were not treated as independent samples. Data are presented as the mean ± standard deviation. Statistical analyses were performed using SPSS Statistics 22.0. Before one-way analysis of variance, data normality was assessed using the Shapiro–Wilk test, and homogeneity of variance was evaluated using Levene’s test. The effect of storage duration was examined by one-way analysis of variance, and mean comparisons were conducted using Duncan’s multiple range test. Statistical significance was accepted at *p* < 0.05. Because no multivariate analysis was performed, the observed variables were interpreted only as parallel temporal changes during frozen storage.

## 3. Results and Discussion

### 3.1. Effects of Frozen Storage on Thawing Behavior and Water-Retention Properties

**Figure 1:**
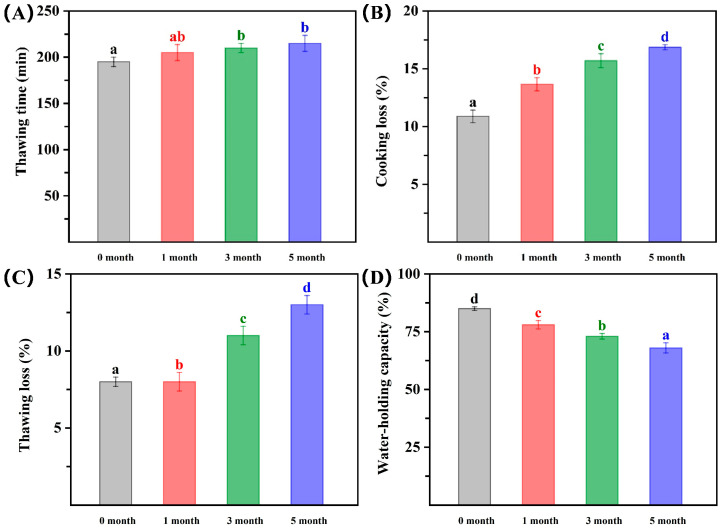
The effects of frozen storage on thawing behavior as well as water-retention properties of giant freshwater prawn (*Macrobrachium rosenbergii*) muscle. (**A**) Thawing time; (**B**) cooking loss; (**C**) thawing loss; and (**D**) water-holding capacity. Values are expressed as the mean ± standard deviation. Different lowercase letters indicate significant differences among storage periods (*p* < 0.05).

Frozen storage significantly affected the thawing behavior and water-retention properties of giant freshwater prawn muscle (Figure 1). Thawing time increased from 190.00 min at 0 months to 213.33 min after 5 months of storage (Figure 1A). The 3- and 5-month groups required significantly longer thawing times than the 0-month group (*p* < 0.05), whereas the value at 1 month was intermediate and did not differ significantly from those at 0 or 3 months. The prolonged thawing time may be associated with storage-induced changes in water distribution, tissue structure, and heat-transfer behavior. Nevertheless, because thawing time is also influenced by sample mass, geometry, and ambient conditions, this result should be interpreted together with the direct water-loss indices.

Cooking loss and thawing loss increased progressively with storage time, whereas water-holding capacity showed the opposite trend. Cooking loss increased from 10.88 ± 0.54% at 0 months to 13.66 ± 0.57%, 15.70 ± 0.61%, and 16.86 ± 0.22% after 1, 3, and 5 months, respectively (Figure 1B). Similarly, thawing loss increased from 8.21 ± 0.29% to 9.43 ± 0.56%, 10.74 ± 0.64%, and 12.51 ± 0.54% over the same storage period (Figure 1C). In contrast, water-holding capacity decreased from 85.45 ± 0.78% at 0 months to 77.70 ± 1.84%, 72.55 ± 1.17%, and 68.46 ± 2.22% after 1, 3, and 5 months, respectively (Figure 1D). Significant differences were observed among all storage periods for these three indices (*p* < 0.05). After 5 months, cooking loss and thawing loss increased by approximately 55.0% and 52.5%, respectively, whereas water-holding capacity decreased by approximately 19.9%. These coordinated changes demonstrate that prolonged frozen storage progressively impaired the ability of prawn muscle to retain water during thawing, centrifugation, and heating.

The deterioration in water retention was plausibly associated with the combined effects of physical tissue damage and changes in muscle proteins. Although ice-crystal morphology was not directly measured in the present study, previous studies have suggested that ice-crystal formation and recrystallization during frozen storage may disrupt cell membranes and muscle-fiber bundles, enlarge inter-fiber spaces, and facilitate the release of intracellular water [5]. Meanwhile, oxidation and denaturation of myofibrillar proteins may reduce the availability of hydrophilic binding sites, weaken protein-water interactions, and impair the ability of the myofibrillar network to immobilize water. Increased thawing loss and progressive disruption of muscle-fiber arrangement have been reported in frozen or repeatedly freeze-thawed shrimp [1,2]. Ji et al. [4] further linked frozen-storage-induced protein oxidation and denaturation to reduced myofibrillar water-holding capacity. In giant freshwater prawn, rapid freezing was more effective than slower freezing methods in limiting thawing loss and preserving muscle quality during frozen storage [3]. However, because the 0-month group represented an initial frozen–thawed control rather than fresh, unfrozen prawn muscle, these results should be interpreted as changes in water-retention properties during prolonged frozen storage after the initial freezing–thawing procedure, rather than as the overall effect of freezing itself.

### 3.2. Changes in Freshness, Oxidation, and Myofibrillar Integrity During Frozen Storage

**Figure 2:**
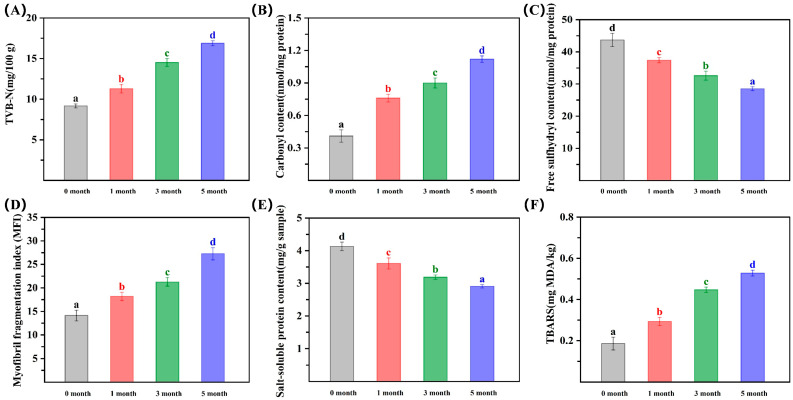
Effects of frozen storage on the freshness, lipid and protein oxidation, and myofibrillar properties of giant freshwater prawn muscle. (**A**) Total volatile basic nitrogen content; (**B**) protein carbonyl content; (**C**) free sulfhydryl content; (**D**) myofibril fragmentation index; (**E**) salt-soluble protein content; and (**F**) TBARS value. Values are expressed as the mean ± standard deviation. Different lowercase letters indicate significant differences among storage periods (*p* < 0.05).

Frozen storage progressively affected the freshness of giant freshwater prawn muscle (Figure 2). TVB-N content increased significantly from 9.17 ± 0.25 mg/100 g at 0 months to 11.29 ± 0.52, 14.52 ± 0.48, and 16.89 ± 0.31 mg/100 g after 1, 3, and 5 months, respectively (Figure 2A; *p* < 0.05). The accumulation of TVB-N reflects the continued degradation of proteins and other nitrogen-containing compounds into volatile basic products. Although freezing substantially suppresses microbial growth and enzymatic activity, it does not completely arrest residual proteolytic and chemical reactions during prolonged storage. Similar storage-dependent increases in TVB-N have been reported in frozen giant freshwater prawn [3]. In marine-trawling shrimp, decreasing the storage temperature from −11 to −37 °C effectively slowed TVB-N accumulation [13]. However, because microbiological analyses were not performed in the present study, the relative contributions of microbial activity and endogenous enzymatic degradation to TVB-N accumulation could not be distinguished. Therefore, TVB-N was interpreted as a freshness-related chemical indicator rather than direct evidence of microbial spoilage.

Protein oxidation was evidenced by the increase in carbonyl content and the simultaneous decrease in free sulfhydryl content. Carbonyl content increased significantly from 0.41 ± 0.06 nmol/mg protein at 0 months to 0.76 ± 0.04, 0.90 ± 0.05, and 1.12 ± 0.03 nmol/mg protein after 1, 3, and 5 months, respectively (Figure 2B; *p* < 0.05). In contrast, free sulfhydryl content decreased from 43.70 ± 2.06 to 37.43 ± 0.82, 32.61 ± 1.37, and 28.54 ± 0.58 nmol/mg protein over the same period (Figure 2C; *p* < 0.05). Protein carbonyls can arise from the direct oxidation of susceptible amino acid side chains and from secondary reactions between proteins and lipid-derived carbonyl compounds [14]. The loss of free sulfhydryl groups likely resulted from the oxidation of cysteine residues into disulfide bonds or other oxidized derivatives, which may subsequently promote protein cross-linking and conformational rearrangement [10,15].

Frozen storage also markedly altered myofibrillar integrity and protein extractability. The myofibril fragmentation index (MFI) increased significantly from 14.13 ± 1.14 at 0 months to 18.20 ± 0.87, 21.27 ± 0.90, and 27.27 ± 1.30 after 1, 3, and 5 months, respectively (Figure 2D; *p* < 0.05). Conversely, salt-soluble protein content decreased from 4.13 ± 0.13 mg/g sample to 3.61 ± 0.17, 3.19 ± 0.07, and 2.91 ± 0.05 mg/g sample (Figure 2E; *p* < 0.05). The nearly twofold increase in MFI indicates progressive fragmentation and loss of integrity of the myofibrillar structure. Similar increases in carbonyl content and MFI have been reported in Pacific white shrimp during partial freezing storage [16]. Moreover, inhibition of endogenous cysteine proteases reduced MFI and alleviated muscle softening in frozen giant freshwater prawn, suggesting that residual proteolysis may contribute to myofibrillar degradation [17]. However, protease activity was not directly measured in the present study; therefore, endogenous proteolysis is discussed only as a plausible mechanism rather than direct experimental evidence. Meanwhile, the approximately 29.5% reduction in salt-soluble protein content suggests decreased protein extractability associated with oxidation, denaturation, and structural modification of muscle proteins [4,10]. Freezing-related physical damage may have further accelerated myofibrillar disruption, although ice-crystal morphology was not directly determined in the present study.

The TBARS value increased significantly from 0.186 ± 0.031 mg MDA/kg at 0 months to 0.293 ± 0.020, 0.447 ± 0.013, and 0.528 ± 0.014 mg MDA/kg after 1, 3, and 5 months, respectively (Figure 2F; *p* < 0.05). The approximately 2.8-fold increase after 5 months indicates the progressive accumulation of secondary lipid oxidation products. As previously reported, ice-crystal-related disruption of cellular membranes and organelles may release oxidative enzymes and endogenous pro-oxidants, thereby facilitating lipid oxidation during frozen storage [1]. Progressive increases in TBARS have also been reported in giant freshwater prawn and marine-trawling shrimp during frozen storage [5,8]. Rapid initial freezing and lower storage temperatures were more effective in limiting lipid oxidation in the respective studies [3,13]. In shrimp surimi products, antioxidant supplementation also retarded TBARS accumulation during frozen storage [10].

The concurrent increases in TVB-N, TBARS, carbonyl content, and MFI, together with decreases in free sulfhydryl and salt-soluble protein contents, demonstrate progressive freshness deterioration, lipid and protein oxidation, and disruption of the myofibrillar structure. Reactive lipid oxidation products may have further contributed to protein carbonylation and cross-linking, although this interaction was not directly measured in the present study. These oxidative and structural changes likely weakened the muscle protein network and reduced its ability to immobilize water, consistent with the reduction in water-holding capacity shown in Figure 1 and the subsequent deterioration in texture and muscle microstructure.

### 3.3. Changes in Textural Properties During Frozen Storage

Frozen storage progressively deteriorated most textural properties of giant freshwater prawn muscle (Table 1). Shear force decreased significantly from 8.43 ± 0.10 N at 0 months to 7.12 ± 0.56 N after 1 month and 5.92 ± 0.19 N after 3 months (*p* < 0.05). Although it further decreased to 5.45 ± 0.44 N after 5 months, no significant difference was observed between the 3- and 5-month groups. Hardness decreased continuously from 955.44 ± 20.40 g at 0 months to 853.62 ± 14.19, 806.07 ± 7.27, and 755.09 ± 13.84 g after 1, 3, and 5 months, respectively, with significant differences among all storage periods (*p* < 0.05). Cohesiveness similarly decreased from 0.45 ± 0.02 to 0.31 ± 0.01. After 5 months, shear force, hardness, and cohesiveness were reduced by approximately 35.3%, 21.0%, and 31.1%, respectively. These reductions indicate a progressive loss of resistance to cutting and compression, together with weakening of the internal bonding within the muscle tissue. Gumminess and chewiness also decreased significantly throughout frozen storage. Gumminess declined from 426.29 ± 23.75 g at 0 months to 230.71 ± 3.77 g after 5 months, while chewiness decreased from 414.84 ± 24.56 to 225.66 ± 6.48 g, corresponding to reductions of approximately 45.9% and 45.6%, respectively. Their pronounced declines were consistent with the simultaneous reductions in hardness and cohesiveness. In contrast, springiness remained between 0.97 and 0.99, with no significant differences among the storage periods (*p* > 0.05). Thus, continuous frozen storage had a greater effect on muscle strength, cutting resistance, and internal cohesion than on the relative recovery of the tissue after compression.

The deterioration in texture likely resulted from the combined effects of water loss, physical disruption of muscle fibers, protein oxidation, and residual endogenous proteolysis. Repeated freeze–thaw treatment has been shown to reduce the textural quality and water-holding capacity of Pacific white shrimp through water migration and myofibrillar protein denaturation [18]. Rapid freezing also preserved the texture and microstructure of the giant freshwater prawn more effectively than slower freezing methods, indicating that the extent of initial ice-crystal damage affects subsequent texture retention [3]. Furthermore, inhibition of endogenous cysteine proteases reduced myofibril fragmentation and alleviated muscle softening in frozen giant freshwater prawn, confirming the contribution of proteolysis to texture deterioration [17]. Similar decreases in hardness and chewiness, accompanied by endogenous protease activity and myofibrillar dissociation, have been observed in sword prawn during frozen storage [19]. In the present study, the decreases in shear force, hardness, cohesiveness, gumminess, and chewiness coincided with increased thawing loss and MFI and with reduced water-holding capacity and salt-soluble protein content. Protein oxidation, as reflected by increased carbonyl content and decreased free sulfhydryl content, may have further weakened the structural and functional integrity of the myofibrillar network. These biochemical and structural changes likely reduced muscle-fiber connectivity and internal tissue cohesion, thereby resulting in progressive softening of giant freshwater prawn muscle during frozen storage.

### 3.4. Histological Changes in Muscle Tissue

**Figure 3:**
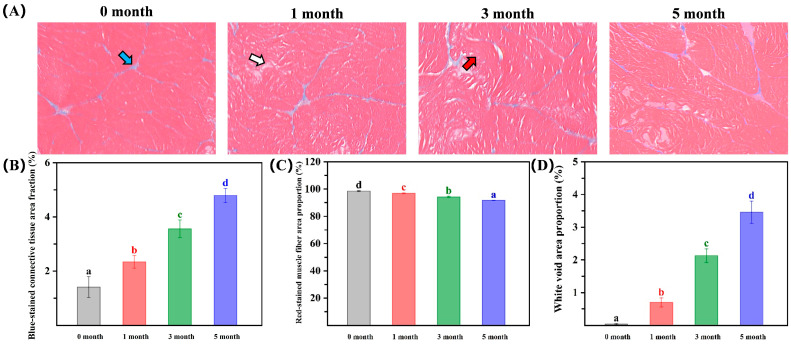
Histological changes in giant freshwater prawn (*Macrobrachium rosenbergii*) during frozen storage. (**A**) Representative Masson’s trichrome-stained sections; (**B**) blue-stained connective tissue area fraction; (**C**) red-stained muscle fiber area fraction; and (**D**) white void area fraction. Muscle fibers are stained red, connective tissue is stained blue, and unstained white regions mainly indicate voids or intermuscular gaps; colored arrows (blue, white, and red) indicate representative intermuscular gaps. Values are expressed as the mean ± standard deviation of three independent biological replicates. For each replicate, the value was calculated as the average of three non-overlapping microscopic fields. Different lowercase letters indicate significant differences (*p* < 0.05).

Masson’s trichrome staining revealed progressive disruption of giant freshwater prawn muscle during frozen storage (Figure 3A). At 0 months, the muscle tissue exhibited a relatively compact and continuous structure, with closely arranged red-stained muscle fibers and thin blue-stained connective tissue distributed between the fiber bundles. Slight fiber separation and small inter-fiber gaps appeared after 1 month. At 3 and 5 months, the muscle fibers became increasingly irregular, wavy, and loosely arranged, accompanied by enlarged inter-fiber spaces and localized tissue disruption. These morphological changes indicate a gradual loss of muscle structural integrity with increasing storage time. Quantitative image analysis confirmed the histological deterioration. The blue-stained connective-tissue area fraction increased significantly from 1.41 ± 0.38% at 0 months to 2.34 ± 0.23%, 3.56 ± 0.33%, and 4.79 ± 0.26% after 1, 3, and 5 months, respectively (Figure 3B; *p* < 0.05). In contrast, the red-stained muscle-fiber area fraction decreased from 98.55 ± 0.39% to 96.96 ± 0.37%, 94.30 ± 0.45%, and 91.76 ± 0.08% over the same period (Figure 3C; *p* < 0.05). Meanwhile, the white void area fraction increased markedly from 0.04 ± 0.02% at 0 months to 0.70 ± 0.14%, 2.13 ± 0.21%, and 3.46 ± 0.34% after 1, 3, and 5 months, respectively (Figure 3D; *p* < 0.05). The relative blue-stained connective tissue area increased during frozen storage. This apparent increase may be mainly attributed to muscle-fiber shrinkage, fiber separation, and changes in tissue organization. Because these fractions were expressed relative to the total image area, the increase in the blue-stained fraction should not be interpreted as an actual increase in collagen content. Instead, it likely reflects greater exposure and relative occupancy of pre-existing connective tissue as the muscle fibers shrank, separated, and lost red-stained area.

Comparable structural damage has been observed in other frozen crustaceans. Repeated freeze–thaw cycles caused weakened attachment between muscle fibers and loss of Z-disks in black tiger shrimp and Pacific white shrimp [2]. In giant freshwater prawn, rapid freezing preserved muscle microstructure more effectively than slower freezing methods, indicating that the extent of freezing-induced physical damage influences subsequent structural stability [3]. Endogenous proteases have also been associated with myofibrillar dissociation and muscle-protein degradation in frozen sword prawn [19]. Similarly, repeated freeze–thaw cycles transformed the initially dense and continuous microstructure of cooked crayfish into an irregular and disrupted structure with enlarged fiber gaps [20]. The observed histological damage may be explained by the combined effects of freezing-related physical stress and biochemical degradation. As suggested by previous studies, ice-crystal formation and recrystallization may exert mechanical stress on cell membranes and muscle fibers, leading to fiber deformation and enlargement of inter-fiber spaces [5]. Simultaneously, protein oxidation, denaturation, and residual endogenous proteolysis may weaken connections within and between muscle fiber bundles [17,19]. The decrease in red-stained muscle area and increase in white void area were consistent with the increased MFI, thawing loss, and cooking loss, and with the reductions in water-holding capacity and textural strength. Thus, the Masson staining results provide direct histological evidence that prolonged frozen storage progressively disrupted the structural integrity of giant freshwater prawn muscle. Nevertheless, the histological observations in the present study mainly reflect structural changes in the selected abdominal muscle region and should not be overgeneralized to the entire muscle tissue. Future studies could include more tissue sections, microscopic fields, and anatomical sampling sites to improve the representativeness of histological evaluation.

### 3.5. Changes in Relative Water-Population Distribution During Frozen Storage

**Figure 4:**
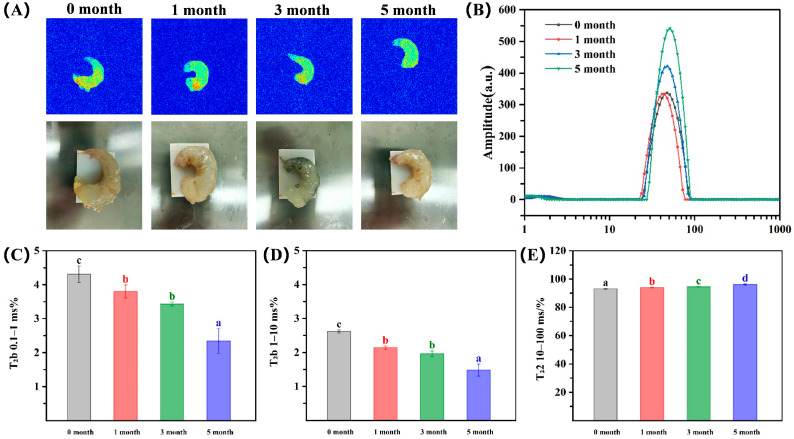
Changes in relative water distribution and water-population characteristics in giant freshwater prawn muscle during frozen storage. (**A**) Proton-density-weighted MRI pseudo-color images; (**B**) T_2_ relaxation-time distributions; (**C**) relative proportion of P_2_b; (**D**) relative proportion of P_2_1; and (**E**) relative proportion of P_2_2. P_2_b, P_2_1, and P_2_2 represent the relative peak-area proportions of their corresponding T_2_ populations. In Figure 4A, the pseudo-colors represent relative proton signal intensity in MRI images, reflecting the relative spatial distribution of water-related signals in prawn muscle. Warmer colors indicate higher signal intensity, whereas cooler colors indicate lower signal intensity. The pseudo-color differences should be interpreted as relative signal-intensity distribution rather than absolute water content. Values are expressed as the mean ± standard deviation. Different lowercase letters indicate significant differences among storage periods (*p* < 0.05).

Changes in the water distribution of giant freshwater prawn muscle over frozen storage were evaluated by low-field nuclear magnetic resonance (LF-NMR) and magnetic resonance imaging (MRI) (Figure 4). The proton-density-weighted pseudo-color images showed progressive changes in the spatial distribution and intensity of water-related signals with increasing storage time (Figure 4A). Because MRI provided primarily qualitative information in the present study, changes in the water populations were evaluated mainly from the quantitative T_2_ relaxation data. All samples exhibited a dominant peak within 10–100 ms (Figure 4B), indicating that the P_2_2 population accounted for most of the detected water throughout storage. The relative peak-area proportion of the short-relaxation P_2_b population decreased from 4.31 ± 0.24% at 0 months to 3.80 ± 0.19%, 3.43 ± 0.06%, and 2.34 ± 0.37% after 1, 3, and 5 months, respectively (Figure 4C). No significant difference was observed between the 1- and 3-month groups, whereas the lowest value occurred after 5 months (*p* < 0.05). Similarly, P_2_1 decreased significantly from 2.62 ± 0.05% at 0 months to 2.14 ± 0.05%, 1.96 ± 0.08%, and 1.48 ± 0.18% after 1, 3, and 5 months, respectively (Figure 4D). The values at 1 and 3 months did not differ significantly, while the 5-month group was significantly lower than the other groups (*p* < 0.05). In contrast, the dominant P_2_2 population increased progressively from 93.07 ± 0.26% to 94.06 ± 0.18%, 94.60 ± 0.02%, and 96.18 ± 0.41% over the same storage period, with significant differences among all groups (Figure 4E; *p* < 0.05). The decreases in P_2_b and P_2_1, together with the increase in P_2_2, indicate a relative redistribution of water populations from short-relaxation components toward the dominant longer-relaxation component. However, P_2_b, P_2_1, and P_2_2 represent relative proportions normalized to the total LF-NMR signal area. Therefore, the increase in P_2_2 should not be interpreted as an increase in absolute moisture content or improved water retention. Moreover, because absolute water contents for each population were not calculated and the relaxation times themselves were not statistically analyzed, the LF-NMR results should be interpreted primarily as changes in the relative water-population distribution rather than as definitive evidence of increased molecular mobility.

Similar water redistribution has been observed in other aquatic products. During partial freezing storage of Pacific white shrimp, the proportions of bound and immobilized water decreased, whereas that of the more mobile water population increased as storage progressed [21]. Repeated freeze–thaw cycles also increased the relaxation times of mobile water populations in Pacific white shrimp and reduced the intensity of MRI pseudo-color images, indicating increased water mobility and redistribution from immobilized to free water [22]. Deng et al. [23] further demonstrated that freezing and thawing conditions markedly affected proton distribution, juice leakage, and protein denaturation in Pacific white shrimp. An increased proportion of relatively mobile water, together with enlarged muscle-fiber gaps and reduced water-holding capacity, was similarly reported in freeze–thawed cuttlefish [24]. The redistribution observed in the present study was likely associated with both physical tissue damage and deterioration of the muscle protein network. Although ice-crystal size and distribution were not directly characterized, freezing-related structural disruption may have reduced the physical restriction of water within the muscle tissue. Meanwhile, oxidation, denaturation, conformational rearrangement, and fragmentation of myofibrillar proteins may reduce the number of accessible hydrophilic sites and weaken protein-water interactions [4]. Consequently, water associated with short-relaxation components may become less tightly retained and more susceptible to release. This interpretation is consistent with the increased thawing and cooking losses, decreased water-holding capacity, and enlarged white void area observed in Figure 1 and Figure 3. Overall, the LF-NMR results indicate that prolonged frozen storage altered the relative distribution of muscle water and weakened the water-retention ability of giant freshwater prawn muscle.

### 3.6. Changes in Protein Secondary Structure During Frozen Storage

**Figure 5:**
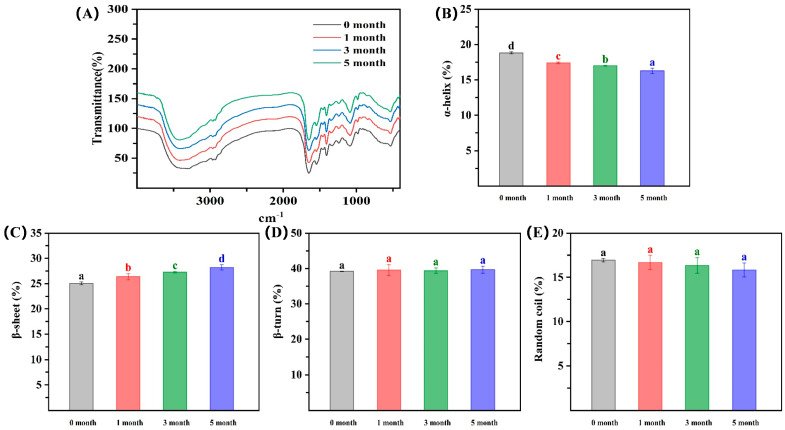
FTIR spectra and protein secondary-structure composition of giant freshwater prawn muscle during frozen storage. (**A**) FTIR spectra; (**B**) α-helix content; (**C**) β-sheet content; (**D**) β-turn content; and (**E**) random-coil content. Results are presented as the mean ± standard deviation. Different lowercase letters denote significant differences among storage times (*p* < 0.05).

Protein secondary-structure alterations in giant freshwater prawn muscle over frozen storage were assessed using Fourier transform infrared spectroscopy (FTIR) (Figure 5). The four groups exhibited similar overall spectral profiles, with no marked appearance of new absorption bands or substantial shifts in the major characteristic peaks (Figure 5A). Nevertheless, variations in the intensity and shape of the amide bands suggested alterations in protein conformation. The amide I region at 1600–1700 cm^−1^, which is dominated by the C=O stretching vibration of peptide bonds, was therefore subjected to curve fitting to determine the relative proportions of the secondary-structure components [25]. The α-helix content decreased progressively from 18.83 ± 0.13% at 0 months to 17.40 ± 0.12%, 17.00 ± 0.06%, and 16.28 ± 0.36% after 1, 3, and 5 months, respectively (Figure 5B; *p* < 0.05). This represented a relative reduction of approximately 13.5% after 5 months. In contrast, the β-sheet content increased from 25.05 ± 0.29% to 26.37 ± 0.63%, 27.27 ± 0.13%, and 28.22 ± 0.52% over the same period (Figure 5C; *p* < 0.05), corresponding to an increase of approximately 12.7%. These coordinated changes indicate a progressive redistribution from α-helical conformations toward a greater relative contribution of β-sheet structures during frozen storage. The β-turn content remained relatively stable, ranging from 39.18 ± 0.03% to 39.66 ± 1.00%, with no significant differences among storage periods (Figure 5D; *p* > 0.05). Similarly, random-coil content varied from 16.93 ± 0.19% to 15.84 ± 0.80%, but their differences were not significant (Figure 5E). Thus, the principal structural changes were characterized by the loss of α-helical conformations and enrichment of β-sheet structures rather than by a generalized increase in disordered conformations.

The decrease in α-helix content may be associated with the disruption of the intramolecular hydrogen bonds that stabilize the helical structure. During frozen storage, ice-crystal-induced dehydration, concentration of solutes in the unfrozen phase, and oxidative modification of amino acid side chains may destabilize the native protein conformation. Partial unfolding can expose previously buried polypeptide regions and facilitate intermolecular association, which may contribute to the increased relative proportion of β-sheet structures. However, because protein aggregation was not directly quantified, this interpretation should be considered as a possible structural consequence rather than direct evidence of aggregation [14,15]. Hu and Xie [25] similarly observed a decrease in α-helix content and an increase in β-sheet content in Trachurus murphyi subjected to repeated freeze–thaw cycles. In cuttlefish, α-helix content also decreased, although the major accompanying change was an increase in random-coil structures [24], indicating that the precise pattern of secondary-structure redistribution may vary among species and freezing conditions. Moreover, multi-frequency ultrasound-assisted freezing maintained a higher α-helix/β-sheet ratio and reduced protein oxidation in large yellow croaker during long-term frozen storage [26]. The reduction in the α-helix level and increase in the content of β-sheet were consistent with the accumulation of protein carbonyls and the decreases in free sulfhydryl and salt-soluble protein contents observed in the present study. These results suggest that oxidative modification and conformational rearrangement were associated with changes in protein secondary structure. Such conformational changes may weaken protein–water interactions and the functional integrity of the myofibrillar network, thereby contributing to the decreases in water-holding capacity and textural strength during frozen storage.

### 3.7. Changes in Protein Tertiary Structure During Frozen Storage

**Figure 6:**
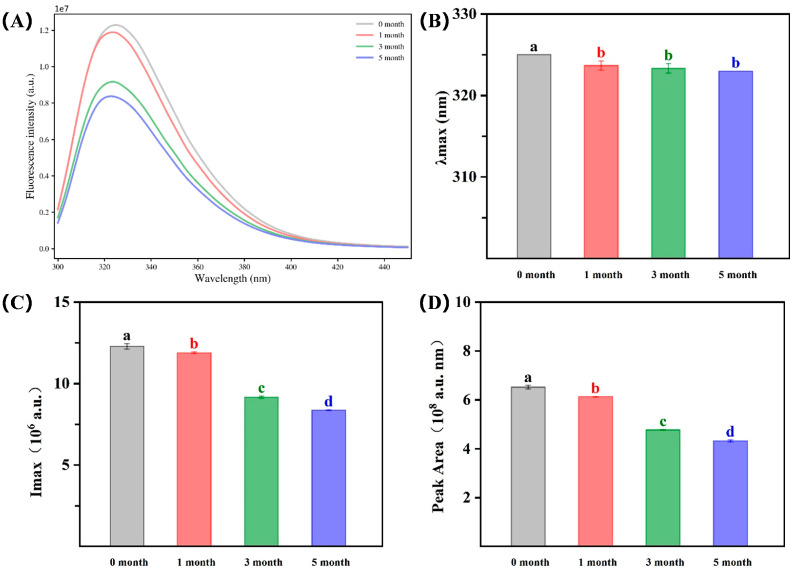
Changes in the intrinsic fluorescence characteristics of giant freshwater prawn muscle proteins during frozen storage. (**A**) Intrinsic fluorescence emission spectra; (**B**) maximum emission wavelength (λ_max); (**C**) maximum fluorescence intensity (I_max); and (**D**) integrated fluorescence peak area. Values are expressed as mean ± standard deviation. Different lowercase letters indicate significant differences among storage periods (*p* < 0.05).

Intrinsic fluorescence spectroscopy was used to evaluate changes in the tertiary conformation of giant freshwater prawn muscle proteins during frozen storage (Figure 6). Upon excitation at 280 nm, protein fluorescence arises mainly from aromatic amino acid residues, with tryptophan generally making the predominant contribution because of its high sensitivity to the surrounding microenvironment [27]. All samples exhibited a characteristic emission maximum at approximately 323–325 nm (Figure 6A). However, the overall fluorescence intensity decreased progressively with increasing storage time, with the lowest intensity observed after 5 months. The maximum emission wavelength (λmax) shifted from 325 nm at 0 months to 323 nm after frozen storage, corresponding to a slight blue shift of approximately 2 nm (Figure 6B). The λmax of the 0-month group was significantly higher than that of the 1-, 3-, and 5-month groups (*p* < 0.05), whereas no significant differences were detected among the frozen-stored groups. A shift toward a shorter emission wavelength generally suggests that the average microenvironment surrounding aromatic residues became less polar or more hydrophobic. In the present study, this small blue shift may reflect storage-induced conformational rearrangement and changes in the microenvironment of tryptophan residues. Nevertheless, given the limited magnitude of the shift, it should be regarded as supporting evidence of tertiary-structure alteration rather than as an independent indication of extensive protein unfolding. The maximum fluorescence intensity (Imax) decreased significantly from 12.29 × 10^6^ a.u. at 0 months to 8.37 × 10^6^ a.u. after 5 months, representing a reduction of approximately 31.9% (Figure 6C). Similarly, the integrated fluorescence peak area decreased from 6.55 × 10^8^ to 4.32 × 10^8^ a.u.·nm, corresponding to a reduction of approximately 34.1% (Figure 6D). Significant differences in both Imax and integrated peak area were observed among all storage periods (*p* < 0.05). Because the protein concentration and measurement conditions were standardized, the progressive fluorescence quenching suggests changes in the chemical state and local environment of aromatic residues. Oxidation of tryptophan residues, interactions with lipid-derived oxidation products, and possible changes in residue accessibility due to conformational rearrangements have all contributed to the reduced fluorescence intensity.

A progressive decrease in intrinsic fluorescence intensity has also been reported in cuttlefish myofibrillar proteins subjected to repeated freeze–thaw cycles and was associated with protein oxidation and tertiary conformational changes [24]. Similarly, Wan et al. [28] observed substantial fluorescence quenching in mirror carp myofibrillar proteins after repeated freeze–thaw treatments. However, their samples exhibited a red shift rather than the slight blue shift observed in the present study. This difference suggests that freezing-related protein damage may expose aromatic residues to a more polar environment in some systems but promote aggregation-related burial in others, depending on species, protein composition, oxidation degree, and freezing conditions. The decrease in fluorescence intensity and peak area was consistent with increases in carbonyl content and β-sheet proportion and with decreases in free sulfhydryl, salt-soluble protein, and α-helix contents. Together, these results indicate that prolonged frozen storage induced oxidative modification and rearrangement of the tertiary conformation of giant freshwater prawn muscle proteins. These changes may have promoted possible intermolecular association, thereby weakening protein–water interactions and the functional integrity of the myofibrillar network.

## 4. Conclusions

Frozen storage at −20 °C progressively deteriorated the quality of giant freshwater prawn muscle. Increased thawing and cooking losses and reduced water-holding capacity were accompanied by lipid and protein oxidation, loss of salt-soluble protein, myofibrillar fragmentation, muscle-fiber separation, and decreases in shear force, hardness, cohesiveness, gumminess, and chewiness. LF-NMR indicated a relative redistribution of water populations from short-relaxation components toward the dominant P_2_2 population, while FTIR and intrinsic fluorescence analyses revealed rearrangements in protein secondary and tertiary structures. These coordinated changes suggested that weakened protein–water interactions and disruption of the myofibrillar network were central to the deterioration of water retention and texture during frozen storage. The present findings suggest that quality-control interventions may warrant particular attention at approximately 3 months of storage under the tested conditions. However, because only four sampling points were included (0, 1, 3, and 5 months) and no statistical breakpoint or change-point analysis was performed, this recommendation should be regarded as a practical reference rather than a statistically confirmed deterioration threshold. A more accurate determination of the critical storage period for frozen giant freshwater prawn will require more frequent sampling around this period. Future studies including a fresh, unfrozen control are needed to further distinguish quality changes caused by initial freezing from those induced by subsequent frozen storage. In addition, because ice-crystal morphology and protease activity were not directly measured in the present study, studies should directly characterize ice-crystal size and distribution, clarify the relative contributions of protein oxidation and endogenous proteolysis, and evaluate phosphate-free water-retention strategies, such as amino acids, antioxidants, and assisted-freezing technologies. Further validation through sensory quality assessment, consumer acceptability testing, microbial analysis for interpreting TVB-N accumulation, and larger-scale storage trials is also needed to improve the industrial applicability of these findings.

## Figures and Tables

**Table 1:** The effects of frozen storage on the textural properties of the giant freshwater prawn.

Frozen Storage Time	Shear Force (N)	Hardness (g)	Springiness	Cohesiveness	Gumminess (g)	Chewiness (g)
0 month	8.43 ± 0.096 c	955.44 ± 20.401 d	0.97 ± 0.029 a	0.45 ± 0.017 d	426.29 ± 23.753 d	414.84 ± 24.564 d
1 month	7.12 ± 0.557 b	853.62 ± 14.191 c	0.99 ± 0.008 a	0.38 ± 0.011 c	328.11 ± 12.25 c	324.05 ± 11.826 c
3 month	5.92 ± 0.187 a	806.07 ± 7.269 b	0.97 ± 0.024 a	0.34 ± 0.015 b	277.09 ± 14.799 b	269.54 ± 10.978 b
5 month	5.45 ± 0.439 a	755.09 ± 13.841 a	0.98 ± 0.016 a	0.31 ± 0.01 a	230.71 ± 3.773 a	225.66 ± 6.48 a

Data are reported as the mean ± standard deviation. Within each column, different lowercase superscript letters denote significant differences among storage times (*p* < 0.05).

## Data Availability

The original contributions presented in the study are included in the article, further inquiries can be directed to the corresponding author.
